# Pre-pregnancy lifestyle characteristics and risk of miscarriage: the Australian Longitudinal Study on Women’s Health

**DOI:** 10.1186/s12884-022-04482-9

**Published:** 2022-03-01

**Authors:** Maria C. Magnus, Richard L. Hockey, Siri E. Håberg, Gita D. Mishra

**Affiliations:** 1 Centre for Fertility and Health, Norwegian Institute of Public Health, P.O. Box 222, Skøyen, 0213 Oslo, Norway; 2 MRC Integrative Epidemiology Unit at the University of Bristol, Bristol, UK; 3 Population Health Sciences, Bristol Medical School, Bristol, UK; 4 School of Public Health, University of Queensland, Brisbane, Australia

**Keywords:** Miscarriage, Alcohol, Body-mass index, Smoking

## Abstract

**Background:**

Previous studies of lifestyle characteristics and risk of miscarriage have mostly been retrospective and failed to account for induced abortions. We examine whether pre-pregnancy body-mass index, alcohol intake and smoking influence the risk of miscarriage after accounting for induced abortions.

**Methods:**

We conducted a prospective cohort study of 9213 women with 26,594 pregnancies participating in the Australian Longitudinal Study on Women’s Health. We examined whether body-mass index, smoking and alcohol intake prior to pregnancy was associated with miscarriage. We estimated adjusted relative risks (RR) using generalized estimating equations with an exchangeable correlation matrix. We explored the impact of accounting for induced abortion by first excluding all induced abortions, and secondly including 50% of induced abortions in the comparison group.

**Results:**

Of the 26,592 pregnancies which occurred during the follow-up period, 19% ended in a miscarriage. We observed an increased risk of miscarriage according to pre-pregnancy obesity compared to normal weight (adjusted RR 1.13; 95% CI 1.05, 1.21), smoking between 10 and 19 cigarettes per day compared to not smoking (adjusted RR 1.13; 95% CI 1.02, 1.25), but not smoking 20 or more cigarettes per day (adjusted RR 1.07; 95% CI 0.94, 1.21) and risky drinking (≥2 units per day; adjusted RR 1.15; 95% CI 1.03, 1.28) compared to low risk drinking (< 2 units per day). The results for smoking (adjusted RR 1.09 for 10–19 cigarettes per day; 95% CI 0.98, 1.21) was attenuated after including 50% of induced abortions in the comparison group.

**Conclusions:**

We observed a modest increased risk of miscarriage according to obesity and risky alcohol intake prior to pregnancy. There was no evidence of a dose-response relationship with smoking, and the association between smoking and risk of miscarriage was attenuated after accounting for induced abortions.

**Supplementary Information:**

The online version contains supplementary material available at 10.1186/s12884-022-04482-9.

## Background

The risk of miscarriage is about 12–15% in recognized pregnancies [1–5]. It is estimated that between 50 and 60% of miscarriages are due to genetic abnormalities [6]. Less is known about the contribution of modifiable lifestyle characteristics in relation to the risk of miscarriage [7]. This is important to clarify, to understand whether targeted interventions before pregnancy aimed at specific lifestyle factors could reduce the likelihood of miscarriage.

A recent meta-analysis of 25 studies supported a higher mean body-mass index (BMI) (adjusted difference 0.7 kg^2^/m; 95% CI: 0.2, 1.3) among women with a history of recurrent miscarriage (*n* = 3822) compared to controls (*n* = 4083) [8]. Furthermore, evidence from 30 cohort studies (265,760 women) support a positive association between both obesity (relative risk (RR) 1.21, 95% CI 1.15, 1.27) and underweight (RR 1.08, 95% CI 1.05, 1.11) with risk of miscarriage [9], highlighting the possibility of a nonlinear relationship between BMI and miscarriage risk. A meta-analysis of 50 studies (> 2 million women) also suggested a modest increased risk of miscarriage among women who were active smokers during pregnancy (RR 1.23, 95% CI 1.16, 1.30) [10]. Another meta-analysis of 24 studies (231,808 women) indicated that women who consumed alcohol during first trimester had an increased risk of miscarriage (OR 1.19, 95% CI 1.12, 1.28) [11].

There is a substantial heterogeneity across the previous studies in design, recruitment strategy for participants and multivariable adjustment strategy. Most of the previous studies examining these lifestyle characteristics in relation to the risk of miscarriage were retrospective studies. The few prospective studies recruited women with a prior history of recurrent miscarriages which opens up for recall and selection bias. It is therefore important to gain additional information from large prospective studies. Furthermore, these studies excluded all pregnancies resulting in induced abortions, which could have biased associations between any risk factors of interests and miscarriage, as a proportion of induced abortion should be included in the comparison group to account for the fact that a proportion of induced abortion would have resulted in miscarriage if the pregnancy had not been terminated [12, 13]. The only exception was one prospective study using information collected in the Danish National Birth Cohort, which was able to account for induced abortions [14]. However, this study evaluated caffeine intake and therefore did not present multivariable adjusted results for smoking. Women who undergo induced abortions show more risk-seeking behaviors, including for example smoking and alcohol intake [15, 16]. Thus, previous studies which mostly excluded induced abortions could have overestimated the associations between these lifestyle characteristics and the risk of miscarriage. It is estimated that approximately half of pregnancies in Australia are unplanned, and that have of these unplanned pregnancies result in terminations [17].

The Australian Longitudinal Study on Women’s Health recruited women born between 1973 and 1978 and were between 18 and 23 years of age when they were recruited in 1996. These women have been followed prospectively at regular intervals, and many have experienced multiple pregnancies since recruitment. They therefore constitute a pre-conception cohort. The objective of this study was to examine whether pre-pregnancy BMI, alcohol intake and smoking influenced the risk of miscarriage after accounting for induced abortions among these women.

## Methods

### Australian Longitudinal Study on Women’s Health

A total of 12,247 women born between 1973 and 1978 participated in the Australian Longitudinal Study on Women’s Health in 1996, when they were between 18 to 23 years of age [18]. The women invited to participate in the cohort constituted a random sample of all women registered in the Medicare database. This prospective cohort study includes individuals receiving benefits from the Medical Benefits Scheme which subsidizes visits to general practitioners and specialists, and the Pharmaceutical Benefits Scheme which subsidizes costs of medications. Since recruitment, there have been eight surveys with response rates ranging from 69 to 42%. However, because women who miss surveys are invited to participate in subsequent surveys, the overall cohort survey response rate has remained relatively stable at around 80% [18].

### Lifestyle characteristics

The women self-reported their height (in cm), weight (in kg), smoking (smoking status, smoking intensity and age at smoking initiation and cessation) and alcohol intake at recruitment and at each follow-up time. Height and weight was used to calculate BMI (weight in kg/height in m^2^), and was categorized as underweight (< 18.5), normal weight (18.5–24.9; reference), overweight (25.0–29.9) or obese (30 or higher). We defined women according to whether they were never smokers (reference), former smokers, smoked less than 10 cigarettes per day, smoked between 10 and 19 cigarettes per day, smoked 20 or more cigarettes per day or were current smokers but the amount was unknown. We also defined women according to whether they were non-drinkers, low-risk drinkers (< 2 units per day; reference), or risky drinkers (≥2 units per day). Risky drinking was defined according to National and Medical Research Council Australian Alcohol Guidelines [19]. One unit of alcohol in Australia contains 10 g of alcohol. We evaluated pre-pregnancy lifestyle factors by examining the lifestyle measures collected in the previous questionnaires when then women was found to have had new pregnancies between two follow-up questionnaires.

### Information on pregnancy history

Women reported their absolute number of deliveries (live and stillbirths), terminations and miscarriages at each follow-up time. We relied on the woman’s report of whether a pregnancy ended in a miscarriage or a stillbirth, as no information on gestational week was available to further distinguish between the two pregnancy outcomes. This information was used to identify new pregnancies between two follow-up times by subtracting the number reported in the later with the previous follow-up time. If a woman did not respond to a specific follow-up questionnaire, we used closest follow-up available. For women who reported a smaller number of total pregnancies at a later time point than the prior time points, we assigned them the total number of pregnancies in the prior time point. We subsequently created a structured dataset containing the total number of new pregnancies women had experienced between recruitment and the end of follow-up. We compared miscarriages to all live births, stillbirths and induced abortions. As we did not have information available on the gestational week of the miscarriages, and we could therefore not separate early/first trimester miscarriages and late miscarriages. We did not include stillbirths as a separate outcome due to insufficient numbers which did not allow for any meaningful analysis. As we did not know the year of birth of all pregnancies that occurred between two follow-up times, we could not study sequential/recurrent miscarriage between two time points, which is commonly defined as two or more subsequent miscarriages without any other pregnancies in between [20].

### Covariates

We obtained information on background characteristics that could influence both the lifestyle characteristics of interest and the risk of miscarriage. These included the age of the woman, marital status (married/de facto versus other), area of residence (major cities, inner regional, outer regional, remote/very remote), managing on income (impossible, difficult always, difficult sometimes, not too bad, it is easy), educational level (less than year 12, year 12 or equivalent, certificate/diploma, university), occupation (manager/professional, semi-skilled, unskilled, not in the labour force), and the number of previous pregnancies (0, 1, 2 or more). Information on these characteristics were taken from the same follow-up questionnaire as the measure of pre-pregnancy lifestyle characteristics.

### Statistical analysis

We examined the relationship between pre-pregnancy BMI, smoking and alcohol intake and miscarriage using generalized estimating equations, with an exchangeable correlation structure, reporting relative risks (RR) and 95% confidence intervals (CI). This model accounts for the correlation between multiple pregnancies to the same woman. Missing information on both exposures and covariates were imputed using the fully conditional specification method, imputing a total of 20 datasets. Results were subsequently pooled using Ruben’s rules [21]. We also examined the shape of the relationship between BMI and risk of miscarriage using restricted cubic splines with 5 knot points. The multivariable regression model adjusted for all mentioned background characteristics. We also mutually adjusted for the pre-pregnancy lifestyle factors examined as exposures in a second multivariable model.

When examining risk factors for miscarriage it is important to account for induced abortions. We know that a proportion of induced abortion would have resulted in a miscarriage if the pregnancy had been allowed to continue. As several exposures (e.g. smoking, alcohol) may be more common among women with induced abortions, excluding these women from the comparison group may lead to overestimations of associations between the lifestyle factors and risk of miscarriage. On the other hand, including all induced abortions in the comparison group might result in an underestimation of associations. The exact proportion of induced abortions that should be included to correct for this potential bias has therefore been a topic of debate and might vary from country to country [12, 13]. We present results both excluding all induced abortions from the comparison group, and including a random sample of 50% of induced abortions in the comparison group [13], to show the plausible range of the estimated association that our data is compatible with. The rationale for including 50% of induced abortions in the comparison group when examining risk factors for miscarriage has previously been described [13]. We repeatedly sampled the 50% of the induced abortions 100 times and used bootstrapping techniques to obtain the combined estimate and standard errors across the iterations. We also explored sensitivity analyses further adjusting for the number of years between when the information on the lifestyle characteristics and the pregnancy outcome was obtained.

All analyses were conducted using SAS version 9.4 (SAS, Research Triangle Park).

## Results

**Fig. 1:**
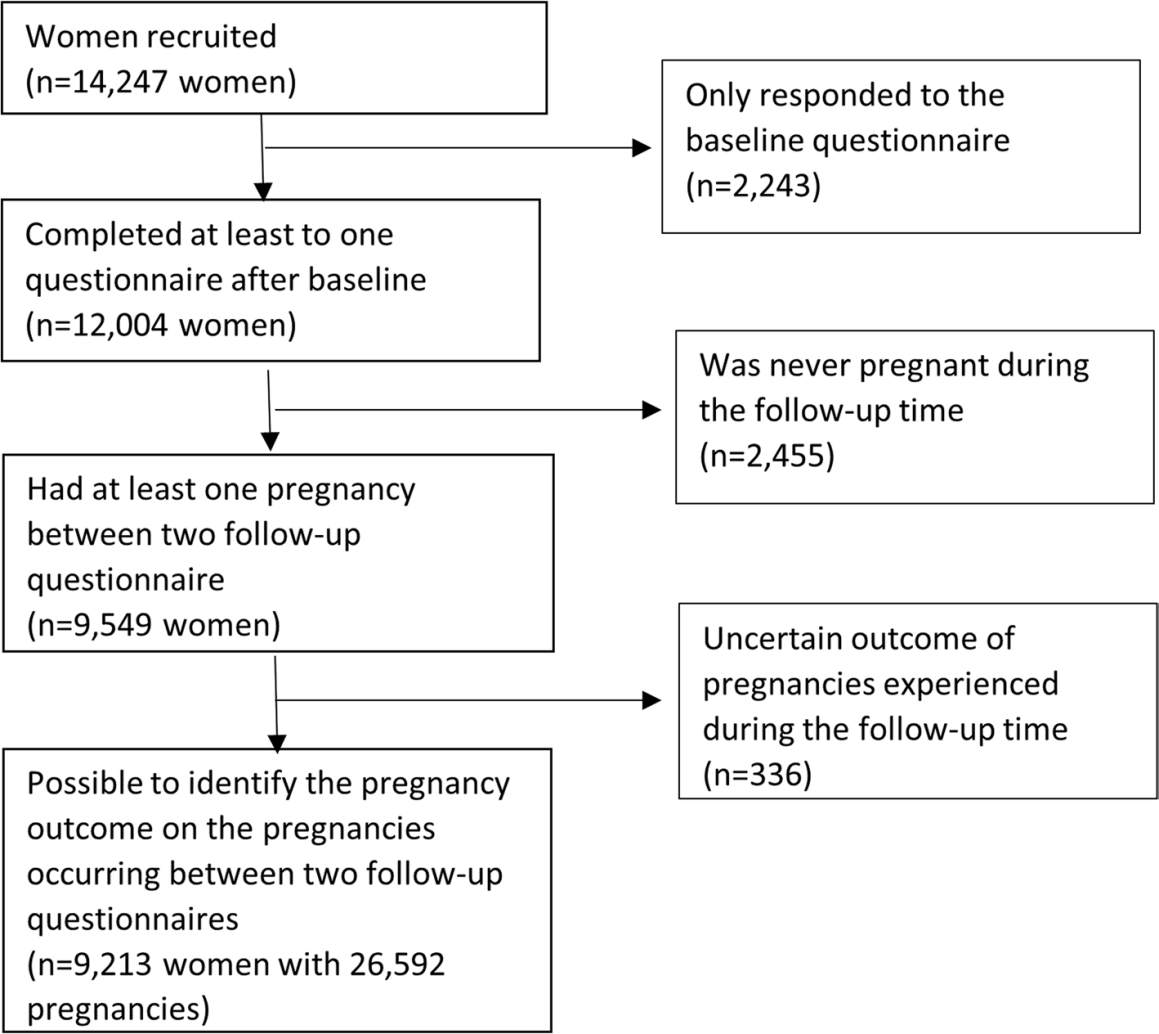
Study population

Of the 14,247 women participating in the cohort, 9549 (67%) had at least one pregnancy during the follow-up period (Fig. 1). After restricting to women with pregnancies for which we could identify the outcome, we included 9213 women with 26,592 pregnancies in the analyses (Fig. 1). Of these pregnancies, 18,031 (67.8%) ended in a live birth, 155 (0.6%) ended in a stillbirth, 3089 (11%) ended in an induced abortion, and 5317 (19%) ended in a miscarriage. Only ~ 3% of pregnancies ended in an induced abortion for medical reasons. The overwhelming majority of the induced abortions were therefore elective.

The distribution of background and lifestyle characteristics according to these pregnancy outcomes are shown in Table 1. Women who experienced a miscarriage were older, more likely to live in major cities, and had a higher number of previous pregnancies compared to women who had live or stillbirths (Table 1). On the other hand, women who had induced abortions were substantially younger, had a lower educational level, were less likely to report managing on their income and were more likely to have semi-skilled or unskilled jobs compared to both women who had miscarriages and those who had a live or stillbirth (Table 1). Women with induced abortions smoked more, were more likely to be risky drinkers, but had lower BMI compared to both women with live births and women with miscarriages. Women with miscarriages had higher BMI, smoked more, and were more likely to be risky drinkers compared to women with live births (Table 1).

**Table 1:** Distribution of background characteristics by pregnancy outcome

	Live or stillbirth (*N* = 18,186)	Miscarriage (*N* = 5317)	Induced abortion (*N* = 3089)	*P*-value
**Age, Mean(SD)**	27.9 (5.6)	28.5 (6.4)	25.5 (6.3)	< 0.001
**Marital status, N(%)**				< 0.001
Married or de facto	13,969 (76.8)	3678 (69.2)	1390 (45.0)	
Other	4139 (22.8)	1620 (30.5)	1687 (55.0)	
Missing	78 (0.4)	19 (0.4)	12 (0.4)	
**Area of residence, N(%)**				0.002
Major cities	9560 (52.6)	2892 (54.4)	1687 (54.6)	
Inner regional	4982 (27.4)	1471 (27.7)	857 (27.7)	
Outer regional	2759 (15.2)	741 (13.9)	404 (13.1)	
Remote/very remote	604 (3.3)	139 (2.6)	102 (3.3)	
Missing	281 (1.5)	74 (1.4)	39 (1.3)	
**Managing on income, N(%)**				< 0.001
Impossible	352 (1.9)	127 (2.4)	95 (3.1)	
Difficult always	2118 (11.6)	656 (12.3)	525 (17.0)	
Difficult sometimes	5543 (30.5)	1478 (27.8)	964 (31.2)	
Not too bad	6246 (34.4)	1783 (33.5)	867 (28.1)	
It is easy	2364 (13.0)	749 (14.1)	350 (11.3)	
Missing	1563 (8.6)	624 (11.7)	288 (9.3)	
**Educational level, N(%)**				< 0.001
Less than year 12	2111 (11.6)	598 (11.2)	478 (15.5)	
Year 12 or equivalent	4191 (23.0)	1291 (24.3)	1127 (36.5)	
Certificate/diploma	4204 (23.1)	1254 (23.6)	680 (22.0)	
University	7441 (40.9)	2117 (39.8)	754 (24.4)	
Missing	239 (1.3)	57 (1.1)	50 (1.6)	
**Occupation, N(%)**				< 0.001
Manager/professional	8022 (44.1)	2436 (45.8)	1283 (41.5)	
Semi skilled	5538 (30.5)	1619 (30.4)	1100 (35.6)	
Unskilled	1143 (6.3)	338 (6.4)	230 (7.4)	
Not in labour force	3115 (17.1)	817 (15.4)	408 (13.2)	
Missing	368 (2.0)	207 (3.9)	68 (2.2)	
**Number of previous pregnancies, N(%)**				< 0.001
0	7100 (39.0)	1018 (19.1)	1095 (35.4)	
1	5748 (31.6)	1314 (24.7)	639 (20.7)	
2+	5338 (29.4)	2985 (56.1)	1355 (43.9)	
**Smoking status, N(%)**				< 0.001
Never smoker	10,190 (56.0)	2716 (51.1)	1159 (37.5)	
Former smoking	4349 (23.9)	1315 (24.7)	679 (22.0)	
Less than 10 cigarettes per day	1332 (7.3)	428 (8.0)	411 (13.3)	
Between 10 and 19 cigarettes per day	989 (5.4)	381 (7.2)	332 (10.7)	
20 or more cigarettes per day	626 (3.4)	234 (4.4)	223 (7.2)	
Current smoker but unknown number of cigarettes per day	445 (2.4)	157 (3.0)	189 (6.1)	
Missing	255 (1.4)	86 (1.6)	96 (3.1)	
**Body-mass index, N(%)**				< 0.001
Underweight	751 (4.1)	246 (4.3)	172 (5.6)	
Normal weight	9602 (52.8)	2772 (52.1)	1731 (56.0)	
Overweight	3534 (19.4)	1120 (21.1)	533 (17.3)	
Obese	2045 (11.2)	794 (14.9)	316 (10.2)	
Missing	2254 (12.4)	385 (7.2)	337 (10.9)	
**Alcohol intake, N(%)**				< 0.001
Low risk drinker	15,307 (84.2)	4508 (84.8)	2634 (85.3)	
Non-drinker	2172 (11.9)	509 (9.6)	196 (6.3)	
Risky drinker	584 (3.2)	259 (4.9)	233 (7.5)	
Missing	123 (0.7)	41 (0.8)	26 (0.8)	

**Fig. 2:**
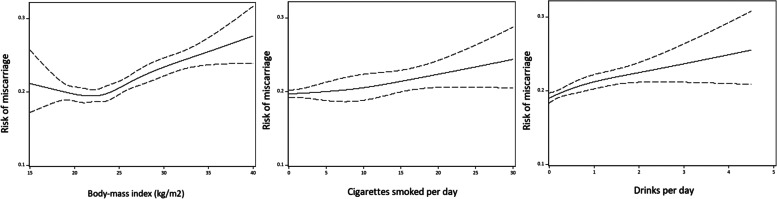
Smoothed plot of the relationship between body-mass index, smoking and frequency of alcohol intake with the risk of miscarriage

We first present the results where we excluded pregnancies ending in induced abortions. There was some evidence of a nonlinear relationship between BMI and risk of miscarriage when we examined the shape of the relationship using restricted cubic splines, while the relationship seemed more linear for smoking and alcohol intake (Fig. 2). We observed an increased risk of miscarriage according to pre-pregnancy obesity (adjusted RR 1.13, 95% CI 1.05, 1.21), smoking between 10 and 19 cigarettes per day (adjusted RR 1.13, 95% CI 1.02, 1.25), but not smoking 20 or more cigarettes per day (adjusted RR 1.07, 95% CI 0.94, 1.21), and risky drinking (adjusted RR 1.15, 95% CI 1.03, 1.28) (Table 2). We also observed a modest decreased risk of miscarriage among women who were non-drinkers (adjusted RR 0.82, 95% CI 0.75, 0.89) (Table 2). Adjustment for the number of previous pregnancies contributed to most of the change between the unadjusted and adjusted results. Further adjustment for different lifestyle characteristics had little impact on results. The results from the complete-case analysis is shown in Additional file 1: Supplementary Table 1, and were largely similar to the multiple imputation results.

**Table 2:** Multiple-imputation analysis of the relationship between body-mass index, smoking and alcohol intake with risk of miscarriage excluding induced abortions (*n* = 23,503)

Exposure	Exposure category	N	N cases (%)	Unadjusted RR (95% CI), *p*-value	Adjusted RR (95% CI)^a^, *p*-value	Adjusted RR (95% CI)^b^, *p*-value
Body-mass index	Underweight	1204	278 (23.1)	1.08 (0.96, 1.22), 0.2	1.07 (0.94, 1.21), 0.3	1.07 (0.94, 1.21), 0.3
	Normal weight	13,505	2918 (21.6)	Ref	Ref	Ref
	Overweight	5506	1253 (22.8)	1.05 (0.98, 1.12), 0.1	1.02 (0.96, 1.09), 0.5	1.03 (0.96, 1.09), 0.4
	Obese	3288	868 (26.4)	1.22 (1.14, 1.32), < 0.001	1.13 (1.05, 1.21), 0.001	1.14 (1.06, 1.23), < 0.001
Smoking	Never smoker	13,071	2749 (21.0)	Ref	Ref	Ref
	Former smoker	5712	1326 (23.2)	1.10 (1.03, 1.17), 0.003	0.99 (0.94, 1.06), 0.9	0.98 (0.92, 1.04), 0.5
	< 10 cigarettes per day	1814	442 (24.4)	1.16 (1.05, 1.28), 0.003	1.02 (0.93, 1.13), 0.6	1.00 (0.91, 1.10), 0.9
	10–19 cigarettes per day	1404	397 (28.3)	1.37 (1.24, 1.51), < 0.001	1.13 (1.02, 1.25), 0.02	1.09 (0.98, 1.21), 0.1
	20 or more cigarettes per day	889	244 (27.4)	1.34 (1.19, 1.51), < 0.001	1.07 (0.94, 1.21), 0.3	1.03 (0.91, 1.17), 0.6
	Unknown amount of smoking	613	159 (25.9)	1.30 (1.13, 1.50), < 0.001	1.06 (0.90, 1.25), 0.5	1.02 (0.86, 1.22), 0.8
Alcohol intake	Low risk drinker	19,957	4544 (22.8)	Ref	Ref	Ref
	Non-drinker	2700	513 (19.0)	0.81 (0.74, 0.89), < 0.001	0.82 (0.75, 0.89), < 0.001	0.81 (0.74, 0.89), < 0.001
	Risky drinker	846	260 (30.7)	1.36 (1.22, 1.52), < 0.001	1.15 (1.03, 1.28), 0.02	1.13 (1.01, 1.27), 0.04

^a^ Adjusted for age, marital status, educational level, area of residence, occupation, managing on income and number of previous pregnancies

^b^ Adjusted for age, marital status educational level, area of residence, occupation, managing on income, number of previous pregnancies, in addition to the other lifestyle factors of interest (body-mass index, smoking and alcohol intake)

Secondly, we included 50% of pregnancies ending in an induced abortions in the comparison group, the association between smoking 10–19 cigarettes per day and risk of miscarriage was attenuated (adjusted RR 1.09, 95% CI 0.98, 1.21) (Table 3). We still observed an increased risk of miscarriage among women who were obese (adjusted RR 1.14, 95% CI 1.07, 1.24), weak evidence of an increased risk of miscarriage among risky drinkers (adjusted RR 1.13, 95% CI 1.01, 1.27) and a lower risk of miscarriage among non-drinkers (adjusted RR 0.84, 95% CI 0.77, 0.92) (Table 3). Only minor changes were observed after mutual adjustment of the lifestyle factors examined. The results from the complete-case analysis is shown in Additional file 1: Supplementary Table 2.

**Table 3:** Multiple-imputation analysis of the relationship between body-mass index, smoking and alcohol intake with risk of miscarriage including 50% of induced abortions (*n* = 26,592)

Exposure	Exposure category	N	N cases (%)	Unadjusted RR (95% CI), *p*-value	Adjusted RR (95% CI) ^a^, *p*-value	Adjusted RR (95% CI) ^b^, *p*-value
Body-mass index	Underweight	1412	278 (19.7)	1.06 (0.94, 1.20), 0.3	1.05 (0.93, 1.19), 0.4	1.05 (0.93, 1.19), 0.4
	Normal weight	15,383	2918 (19.0)	Ref	Ref	Ref
	Overweight	6140	1253 (20.4)	1.06 (1.00, 1.14), 0.07	1.04 (0.97, 1.10), 0.3	1.04 (0.97, 1.10), 0.3
	Obese	3657	868 (23.7)	1.24 (1.15, 1.34), < 0.001	1.15 (1.07, 1.24), < 0.001	1.16 (1.08, 1.25), < 0.001
Smoking	Never smoker	14,267	2749 (19.3)	Ref	Ref	Ref
	Former smoker	6400	1326 (20.7)	1.09 (1.02, 1.16), 0.01	0.98 (0.93, 1.05), 0.6	0.97 (0.91, 1.03), 0.3
	< 10 cigarettes per day	2247	442 (19.7)	1.09 (0.98, 1.20), 0.1	0.98 (0.88, 1.08), 0.6	0.96 (0.87, 1.06), 0.4
	10–19 cigarettes per day	1746	397 (22.7)	1.27 (1.14, 1.41), < 0.001	1.09 (0.98, 1.21), 0.1	1.06 (0.95, 1.18), 0.3
	20 or more cigarettes per day	1120	244 (21.8)	1.23 (1.09, 1.39), 0.001	1.02 (0.90, 1.16), 0.7	0.99 (0.87, 1.13), 0.9
	Unknown amount of smoking	812	159 (19.6)	1.17 (1.01, 1.35), 0.04	1.04 (0.89, 1.22), 0.6	1.02 (0.86, 1.20), 0.8
Alcohol intake	Low risk drinker	22,612	4544 (20.1)	Ref	Ref	Ref
	Non-drinker	2898	513 (17.7)	0.83 (0.76, 0.92), < 0.001	0.84 (0.77, 0.92), < 0.001	0.83 (0.76, 0.91), < 0.001
	Risky drinker	1082	260 (24.0)	1.27 (1.13, 1.42), < 0.001	1.13 (1.01, 1.27), 0.03	1.13 (1.01, 1.27), 0.04

^a^ Adjusted for age, marital status, educational level, area of residence, occupation, managing on income and number of previous pregnancies

^b^ Adjusted for age, marital status, educational level, area of residence, occupation, managing on income, number of previous pregnancies, in addition to the other lifestyle factors of interest (body-mass index, smoking and alcohol intake)

The time between when we obtained information on the lifestyle factors and when the woman reported having been pregnant varied (median 1.5 years). Further adjustment for the number of years between when information on the lifestyle factors and the pregnancy outcome did not change our findings.

## Discussion

Our findings support a modest increased risk of miscarriage according to maternal obesity and risky alcohol consumption. These associations were robust after accounting for induced abortions by including 50% in the comparison group. The observed relationships between smoking prior to pregnancy and risk of miscarriage were attenuated when accounting for induced abortions. Previous studies might therefore have overestimated the associations with smoking.

Important strengths of the current study include the prospective design, where the information on the lifestyle characteristics and pregnancy outcomes were collected independently of each other, our adjustment for a broad range of potential confounding factors, and the possibility to address the impact of including induced abortion in the comparison group. We acknowledge that including 50% of induced abortions might be an over-adjustment, and that the true magnitude of the association might lie somewhere between the estimates obtained excluding and including 50% of induced abortions.

Our study also has some limitations that are worth noting. We cannot exclude the possibility of a selection bias due to the response rate of the cohort. This seems to be reflected in proportion of induced abortions, which is slightly lower than what would be expected, and results in a corresponding higher estimated proportion of miscarriages. We also did not have information on exactly when the women became pregnant, as we only had information on the number of pregnancies the woman had experienced (and their outcomes) at each follow-up time. We used this to identify women who had experienced new pregnancies between two follow-up times. Women might therefore have changed their lifestyle between the time when this information was obtained and when they became pregnant. However, further adjustment for the time between when the information on lifestyle and the identification of the new pregnancy did not change our findings. Despite our adjusting for a broad range of background characteristics, we also can’t exclude a possible role of unmeasured confounding. We were also unable to define recurrent miscarriage based on the information available, and could therefore not examine this as a separate, more sever, outcome. Finally, as we are relying on self-reported information, we are not able to capture unrecognized pregnancies ending in a miscarriage.

We observed an association of a slightly smaller magnitude between obesity and risk of miscarriage (RR 1.15) than reported from previous studies (OR 1.21) [9]. Notably, this meta-analysis of previous studies focused on recurrent miscarriages. In line with what is suspected from previous studies [9], our findings also support that there may be an increased risk of miscarriage among underweight women, but our results were inconclusive and need to be replicated in larger study populations. The relationship we observed between alcohol intake and risk of miscarriage (RR 1.13) was also of a similar magnitude as reported in previous studies (OR 1.19) [11], despite that we are likely overestimating the woman’s alcohol intake during pregnancy. We did not observe an association between smoking and risk of miscarriage after accounting for induced abortions (RR 1.02 for smoking 20 or more cigarettes per day), while previous studies excluding induced abortions reported a modest relationship (RR 1.23 for any smoking during pregnancy) [10]. We note that these previous studies focused on retrospectively reported smoking during pregnancy, while we studied smoking patterns some time prior to pregnancy, and not during pregnancy. We acknowledge that a lot of women quit or reduce their smoking after they find out that they are pregnant. This may be reflected in the lack of an association in our study.

The prevalence of some of the lifestyle factors evaluated have changed in recent decades [22–24]. It is therefore plausible that the relationship between these lifestyle characteristics and the risk of miscarriage might also have changed over time. The women included in this study were born within a relatively narrow time window, and we could therefore not explore this further in the current study.

There are various explanations for why the associations between the lifestyle characteristics examined and risk of miscarriage was modest in our study. As most previous studies gathered information on the lifestyle factors retrospectively, it is possible that their results might be influenced by a differential recall among women with a miscarriage as opposed to women with live births. We are capturing the woman’s lifestyle some time prior to becoming pregnant. Both smoking and alcohol intake during pregnancy has decreased over time [25]. This is likely partly due to women becoming aware that they are pregnant earlier, and subsequently stop drinking and smoking, thereby reducing any potential miscarriage risk with these exposures. On the other hand, the proportion of women of reproductive age who are overweight or obese is increasing [26].

The importance of incorporating induced abortions into studies of risk factors for miscarriage has been debated [3, 12]. We argue that existing studies of the risk of miscarriage according to lifestyle factors have largely ignored this important issue. Excessive alcohol intake and smoking are risk seeking behaviors that are likely more common among women with unplanned pregnancies who end up undergoing induced abortions [27]. Ignoring induced abortions when examining the risk of miscarriage according to lifestyle characteristics could therefore result in overestimated associations. Our results indicate that this may be the case particularly for the relationship between smoking and miscarriage.

A biological plausibility for an influence of both obesity, alcohol intake and smoking on risk of pregnancy loss exists. For example, overweight/obesity is linked to a pro-inflammatory state, while smoking increases the amount of circulating free radicals and results in vasoconstriction, and both lifestyle characteristics are therefore likely to influence placentation and potentially embryo implantation and development [28, 29]. Furthermore, it is hypothesized that smoking and obesity might be part of a common etiology of miscarriage and infertility [30]. This notion is supported by studies linking both obesity and smoking to a decreased likelihood of a successful outcome after use of assisted reproductive technologies [31, 32]. The potential mechanisms underlying a relationship between alcohol consumption and risk of miscarriage is unclear, but might reflect an increased risk of fetal genetic anomalies and epigenetic changes resulting in a higher risk of fetal death [33, 34].

Preconception care is a popular concept but poorly executed [35]. Our findings indicate that there might be a role for giving women who are planning to become pregnant lifestyle advice to mitigate their risk of miscarriage (as well as other adverse pregnancy outcomes). Such advice should be tailored to their specific needs and underlying health. There are obvious health benefits to smoking cessation and weight loss that extend beyond the time that a woman is pregnant. Clinicians should therefore capitalize on the opportunity and provide women with such lifestyle advice as part of their preconception care.

## Conclusions

We observed an increased risk of miscarriage according to smoking, alcohol intake and obesity prior to pregnancy. The associations with smoking was attenuated after accounting for induced abortions. Previous studies excluding induced abortions might therefore have overestimated this association. Clinicians should provide lifestyle guidance as part of the preconception care of women of reproductive age.

## Supplementary Information

**Additional file 1.** Results from complete-case analysis.

## Data Availability

The datasets used and/or analysed during the current study are available from the corresponding author on reasonable request.

## Abbreviations

BMI: Body-mass index
OR: Odds ratio
RR: Relative risk
