# Supplementary material for: Pre-pregnancy lifestyle characteristics and risk of miscarriage: the Australian Longitudinal Study on Women’s Health
Source: BMC Pregnancy Childbirth. 2022 Mar 1;22:169. doi: 10.1186/s12884-022-04482-9 (PMC8887017; doi:10.1186/s12884-022-04482-9)
Supplement: Supplementary file 1 — Additional file 1. Results from complete-case analysis. [file 12884_2022_4482_MOESM1_ESM.docx]

Supplementary Table 1. Complete-case analysis of the relationship between body-mass index, smoking and alcohol intake with risk of miscarriage excluding induced abortions

(n= 19,548)

| Exposure | Exposure category | N (% miscarriage) | Unadjusted RR (95% CI), p-value | Adjusted RR (95% CI)* , p-value | Adjusted RR  (95% CI)± , p-value |
| --- | --- | --- | --- | --- | --- |
| Body-mass index | Underweight | 997 (24.7) | 1.15 (1.02, 1.30), 0.02 | 1.11 (0.98, 1.25), 0.1 | 1.13 (0.99, 1.28), 0.07 |
|  | Normal weight | 12,374 (22.4) | Ref | Ref | Ref |
|  | Overweight | 4,654 (24.1) | 1.08 (1.01, 1.15), 0.02 | 1.04 (0.98, 1.11), 0.2 | 1.04 (0.98, 1.11), 0.2 |
|  | Obese | 2,839 (28.9) | 1.25 (1.16, 1.35), <0.001 | 1.14 (1.06, 1.23), <0.001 | 1.16 (1.07, 1.25), <0.001 |
| Smoking | Never smoker | 12,906 (21.0) | Ref | Ref | Ref |
|  | Former smoker | 5,664 (23.2) | 1.10 (1.03, 1.17), 0.006 | 0.98 (0.93, 1.05), 0.6 | 0.98 (0.92, 1.05), 0.6 |
|  | <10 cigarettes per day | 1,760 (24.3) | 1.18 (1.07, 1.30), 0.001 | 1.03 (0.94, 1.14), 0.5 | 0.99 (0.90, 1.10), 0.9 |
|  | 10-19 cigarettes per day | 1,370 (27.8) | 1.36 (1.22, 1.51), <0.001 | 1.12 (1.00, 1.24), 0.04 | 1.07 (0.96, 1.20), 0.2 |
|  | 20 or more cigarettes per day | 860 (27.2) | 1.32 (1.16, 1.50), <0.001 | 1.05 (0.92, 1.20), 0.4 | 1.06 (0.93, 1.21), 0.4 |
|  | Unknown amount of smoking | 602 (26.1) | 1.31 (1.13, 1.51), <0.001 | 1.06 (0.90, 1.25), 0.5 | 1.02 (0.84, 1.25), 0.8 |
| Alcohol intake | Low risk drinker | 19,815 (22.8) | Ref | Ref | Ref |
|  | Non-drinker | 2,681 (19.0) | 0.80 (0.72, 0.88), <0.001 | 0.81 (0.74, 0.89), <0.001 | 0.83 (0.75, 0.91), <0.001 |
|  | Risky drinker | 843 (30.7) | 1.39 (1.24, 1.55), <0.001 | 1.16 (1.03, 1.29), <0.001 | 1.13 (0.99, 1.30), 0.02 |

*Adjusted for age, marital status, educational level, area of residence, occupation, managing on income and number of previous pregnancies.

± Adjusted for age, marital status educational level, area of residence, occupation, managing on income, number of previous pregnancies, in addition to the other lifestyle factors of interest (body-mass index, smoking and alcohol intake).

Supplementary Table 2. Complete-case analysis of the relationship between body-mass index, smoking and alcohol intake with risk of miscarriage including 50% of induced abortions

(n=22,050)

| Exposure | Exposure category | N (% miscarriage) | Unadjusted RR (95% CI) , p-value | Adjusted RR (95% CI)* , p-value | Adjusted RR  (95% CI)± , p-value |
| --- | --- | --- | --- | --- | --- |
| Body-mass index | Underweight | 1,169 (21.0) | 1.15 (1.02, 1.31), 0.03 | 1.12 (0.98, 1.27), 0.09 | 1.12 (0.98, 1.27), 0.09 |
|  | Normal weight | 14,105 (19.7) | Ref | Ref | Ref |
|  | Overweight | 5,187 (21.6) | 1.10 (1.02, 1.17), 0.008 | 1.05 (0.98, 1.12), 0.1 | 1.05 (0.98, 1.12), 0.1 |
|  | Obese | 3,155 (25.2) | 1.27 (1.18, 1.37), <0.001 | 1.17 (1.08, 1.26), <0.001 | 1.18 (1.09, 1.27), <0.001 |
| Smoking | Never smoker | 14,065 (19.3) | Ref | Ref | Ref |
|  | Former smoker | 6,343 (20.7) | 1.10 (1.03, 1.18), 0.005 | 0.99 (0.92, 1.05), 0.7 | 0.97 (0.91, 1.04), 0.4 |
|  | <10 cigarettes per day | 2,171 (19.7) | 1.09 (0.98, 1.21), 0.1 | 0.97 (0.88, 1.08), 0.6 | 0.95 (0.86, 1.06), 0.4 |
|  | 10-19 cigarettes per day | 1,702 (22.4) | 1.26 (1.12, 1.41), <0.001 | 1.07 (0.96, 1.21), 0.2 | 1.04 (0.92, 1.17), 0.5 |
|  | 20 or more cigarettes per day | 1,083 (21.6) | 1.27 (1.11, 1.46), <0.001 | 1.06 (0.92, 1.22), 0.4 | 1.02 (0.89, 1.17), 0.8 |
|  | Unknown amount of smoking | 791 (19.8) | 1.19 (1.02, 1.39), 0.03 | 1.05 (0.88, 1.26), 0.6 | 1.03 (0.85, 1.23), 0.8 |
| Alcohol intake | Low risk drinker | 22,449 (20.1) | Ref | Ref | Ref |
|  | Non-drinker | 2,877 (17.7) | 0.85 (0.77, 0.94), 0.002 | 0.85 (0.77, 0.93), <0.001 | 0.84 (0.76, 0.92), <0.001 |
|  | Risky drinker | 1,076 (24.1) | 1.31 (1.16, 1.47), <0.001 | 1.16 (1.03, 1.31), 0.01 | 1.16 (1.03, 1.31), 0.02 |

*Adjusted for age, marital status, educational level, area of residence, occupation, managing on income and number of previous pregnancies.

± Adjusted for age, marital status, educational level, area of residence, occupation, managing on income, number of previous pregnancies, in addition to the other lifestyle factors of interest (body-mass index, smoking and alcohol intake).
